# Relationship between time spent playing internet gaming apps and behavioral problems, sleep problems, alexithymia, and emotion dysregulations in children: a multicentre study

**DOI:** 10.1186/s13034-022-00502-w

**Published:** 2022-08-16

**Authors:** Gellan K. Ahmed, Alaa A. Abdalla, Ali M. Mohamed, Lobna A. Mohamed, Hala A. Shamaa

**Affiliations:** 1 Department of Neurology and Psychiatry, Faculty of Medicine, Assiut University, Assiut, Egypt; 2 Department of Neurology and Psychiatry, Faculty of Medicine, Suez Canal University, Ismailia, Egypt; 3 Ministry of Education, Cairo, Egypt; 4 Department of Neurology and Psychiatry, Alexandria University, Alexandria, Egypt; 5 General Secretariat of Mental Health and Addiction Treatment, Demira Mental Health Hospital, Dakahlya, Egypt

**Keywords:** Children, Internet gaming, Alexithymia, Psychiatric, Sleep, Emotion dysregulation

## Abstract

**Background:**

Internet gaming addiction (IGA) is a serious condition that can significantly impact personal and social functioning. Many studies of IGA have been conducted in adolescents and young adults, but there are limited data available in children. We investigated the time spent using internet gaming apps in children and its association with behavioral problems, sleep problems, alexithymia, and emotional regulation.

**Methods:**

The research populations (N = 564) were categorized based on the number of hours spent using online gaming applications. The Strengths and Difficulties Questionnaire, the Children's Sleep Habits Questionnaire Abbreviated, the Children's Alexithymia Measure (CAM), and the Clinical Evaluation of Emotional Regulation–9 were used to assess all participants.

**Results:**

Compared to other groups, children who used internet gaming applications for more than 6 h had a higher proportion of abnormal responses on the emotional symptoms and hyperactivity scales. Children who used internet gaming applications for more than 6 h had the poorest sleep quality (75%), while children who used internet gaming applications for 1–2 h had the best (36.7%). Participants who used internet gaming apps for 1–2 h had significantly lower mean total scores on the emotional regulation scale and total CAM, whereas those using internet gaming apps for more than 6 h had the highest mean scores in the CAM.

**Conclusions:**

Excessive use of internet gaming apps during childhood may be associated with hyperactivity, peer problems, high socioeconomic level, alexithymia concerns, shorter daytime sleep duration, and a delayed morning wake-up.

**Supplementary Information:**

The online version contains supplementary material available at 10.1186/s13034-022-00502-w.

## Background

Games play a critical role in the integration of human behavior and experience. Computer technology has drastically risen in availability and use over the previous two decades, reshaping the leisure world. Beyond social and conventional media, today's young people use the internet and play computer games [1]. In 2018, the International Classification of Diseases (ICD-11) proposed that gaming disorder (GD) should be classified as a disease [2]. Impaired control over gaming, giving gaming a higher priority, and continuing or increasing gaming despite experiencing negative effects are the three symptoms that must be present for GD to meet the diagnostic criteria of (ICD-11) [2]. Thus, the American Academy of Pediatrics recommends that children limit their screen time to 1 to 2 h per day [3]. Similarly, the Canadian Society for Exercise Physiology recommends that children aged 5 to 17 spend no more than 2 h per day on recreational screens [4].

Different rates of IGD have been reported in young people due to different measurement tools and cultural backgrounds. In Europe, for example, the prevalence was 1.2–1.6% [5, 6], whereas in Asia, it ranged from 1.6–18.4% [7–9]. In Egypt, the prevalence of IGD among adolescents was 9.7% [10]. However, more research based on more extensive samples should be undertaken to validate those figures [11].

Excessive video gaming has also been linked to attention problems, poor academic performance [12], anxiety, depressive symptoms, deterioration of interpersonal relationships, family conflicts, youth violence or crime [13], low self-esteem, and dissatisfaction with daily life [14].

Sleep deprivation may result from increased screen time [15, 16]. Delayed bedtimes and late waking times may exacerbate rhythm desynchronization and have a negative effect on academic performance [16]. Psychological stimulation (i.e., an elevated mood caused by social media use) [17], light-emitting screens [18], and decreased sleep duration are all possible causes of disturbed sleep [19]. Players from various time zones frequently participate in multiplayer games [20]. As a result, some gamers may delay logging off or may wake up during the night to continue gaming [5], resulting in inconsistent or chaotic sleep–wake patterns and sleep deprivation. Furthermore, online gaming is associated with increased sleep latency and decreased total rapid eye movement sleep [21].

Gaming and the pursuit of game-related pleasure can cause the neglect of “regular or normal” relationships, school or work-related obligations, and even basic physical requirements. Playing games can thus be viewed as a progression from pleasurable leisure to a problematic and even compulsive habit [22–24]. This can be explained by alexithymia. Alexithymia is characterised by difficulties describing and expressing emotions. Other fundamental characteristics of alexithymia include an externally oriented cognitive style, a constrained imagination, and a lack of empathy [25, 26]. Individuals with alexithymia face significant challenges in forming friendships and typically have low social functioning because of these constraints [27]. The concept that people with alexithymia attempt to manage their emotions through compulsive [28] or impulsive [29] actions has been associated to addictive illnesses. A previous study found that regular gamers exhibited higher levels of alexithymia than irregular gamers [30]. Gaetan et al. proposed that, because alexithymia typically displays with a flat emotional profile and the virtual environment facilitates emotion regulation, online gaming may serve as an attempt to control these characteristics in adolescents with alexithymia [31].In addition, previous research found that adolescents with IGA exhibited more comorbid psychiatric disorders and difficulties expressing emotions, indicating the adoption of avoidance strategies [10].

Furthermore, children and teenagers are thought to use video games as a maladaptive coping mechanism to deal with negative emotions [32]. In this regard, children with limited social skills, the internet in general, and video games, are likely to be preferred over real-life interactions [33, 34]. As a result, emotion avoidance and dysregulation can occur.

Although there have been some studies on problematic internet use and online gaming they have focused mostly on adolescents and young adults, with limited data on children. One reason is that most diagnostic tools are self-administered by patients and can only be done by adolescents. In contrast, there are no parent-administered versions of the GD screen scales that can be utilised with children. Indeed, the diagnostic criteria for GD rely on subjective diagnostic criteria, such as loss of control and continuing or increasing gaming despite suffering negative consequences, which are difficult to assess in children. In order to avoid these problems in the present study we measured the number of hours spent playing online games and asked whether there was any relationship to behavioral problems, sleep problems, alexithymia, and emotional regulation.

## Methods

### Study design, and population

This was a cross-sectional study between February and November of 2021. To cover the various sociodemographic variables, 564 participants ranging in age from 6 to 14 years old were enrolled in three Egyptian cities: Assiut (Upper Egypt), Cairo (central), and Ismailia (lower Egypt). In each city, we choose 3 schools: one governmental school, one private school and one model governmental school (governmental with language modified curriculum). All the schools in the study covered all educational grades (i.e. primary, elementary and secondary school), had the largest number of students in each city, and had an official website. An online link was published on school websites with the agreement of the school's manager to find parents who were willing to participate and evaluate their children. the link consisted of three sections to be answered by parents. The first two sections included information about the student such as age, gender, play on online gaming apps and other information such as a history of substance abuse, psychiatric disorders, or medical conditions (to identify their eligibility for study). The third section included information about the study aims with an option to decline or accept our invitation to join the study. Parents who agreed to our request had their children evaluated at local Child and Adolescent Clinics. Children with an IQ of less than 70 or a history of substance abuse, psychiatric disorders, or medical conditions were not eligible.

About 50 requests to participate were excluded because the online information, such as medical history, was unclear. A total of 680 requests were approved of whom 564 participants eventually entered the study.

The research population (N = 564) was categorized based on the number of hours used for online gaming applications. Group 1: use internet gaming applications for 1–2 h, Group 2: use internet gaming applications for 3–4 h, Group 3: use internet gaming applications for 5–6 while Group 4: uses internet gaming apps for more than 6 h.

The evaluation was done in Arabic by trained psychiatrists and psychologists who interviewed parents about their children. Regarding psychiatric interviews and scores of scales of this study, children who had problems reported to the parent to schedule an appointment with a psychiatric counsellor at school for further evaluation and treatment.

### Tools

#### Demographic data

The information was collected from parents regarding sociodemographic status and the child’s clinical, and medical histories. Information included: age, gender, birth order, number of children, speech delay, motor development, family history of psychiatric problems, previous medical conditions, the number of devices used to access internet gaming apps, and the number of hours spent on internet gaming.

#### The Strengths and Difficulties Questionnaire (SDQ) parent version parent [35]

This consists of 25 questionnaires used to screen for behavioral problems in children aged 4–17 years. It was categorized into five subscales: emotional symptoms, conduct problems, hyperactivity/inattention symptoms, peer relationship problems, and prosocial behavior. The total difficulty score is calculated by adding the first four numbers. Research on the reliability of the SDQ has produced mixed results. However, most studies report internal consistency of the total difficulties score and the subscales scores with a Cronbach α above 0.70 [36, 37].

#### Socioeconomic scale

The educational level of the parents and mothers, their occupation, total family income, and the family's lifestyle were included in this study. Based on their total (raw) score, which is calculated using an equation based on these four characteristics, individuals in a sample are classified as high, middle, or low class. Cronbach’s alphas of SEC is 0.89 [38].

#### The Children’s Alexithymia Measure (CAM) [39]

The Children’s Alexithymia Measure (CAM) is intended to be completed by a parent. The CAM comprises 14 components rated in a range of 0 to 3. Total scores can vary from 0 to 42, with higher numbers indicating more alexithymia.The internal consistency of CAM is coefficient alpha = 0.92 [39]

#### The Children's Sleep Habits Questionnaire Abbreviated (CSHQ-A) [40]

In this study, the Children's Sleep Habits Questionnaire (CSHQ-A) was used to assess sleeping difficulties. These questionnaires were collected retrospectively, with parents recalling sleep patterns, disturbances, or activities from the previous week (e.g., bedtime, sleep behavior, waking during the night, morning wake up). A CSHQ-A score of more than 30 was considered abnormal and indicative of sleep difficulties. It has sensitivity 89.2% and specificity 44.6% [41].

#### The Clinical Evaluation of Emotional Regulation–9

This consists of nine questions derived from Swanson Nolan and Pelham (SNAP-IV) items and applied to a factor of emotional dysregulation. It is used to evaluate emotional regulation and answered by parent. Previously, these items were graded on a 0 to 3 Likert scale, with the extremes of not at all and very much serving as anchors. The highest accuracy for identifying children and adolescents with current significant emotional regulation problems is a score of 4 or higher. The Cronbach’s alphas of the nine retained items were 0.80 [42].

### Statistical analysis

SPSS was used for statistical analysis (version 26). The data was described using frequencies and percentages. To investigate categorical variables, the chi-squared test was performed. If there were statistically significant differences in mean values between more than two groups, the ANOVA test was used. Linear regression was performed to investigate potential risk variables for increased use of internet gaming apps. A p-value of less than 0.05 was considered statistically significant.

## Results

### Demographic characteristics

The research populations (N = 564) were categorized into four groups based on the number of hours used by online gaming application participants. Group 1 uses internet gaming applications for 1–2 h (N = 244), Group 2 uses internet gaming applications for 3–4 h (N = 120), Group 3 uses internet gaming applications for 5–6 (N = 92) while Group 4 uses internet gaming apps for more than 6 h (N = 32).

**Fig. 1:**
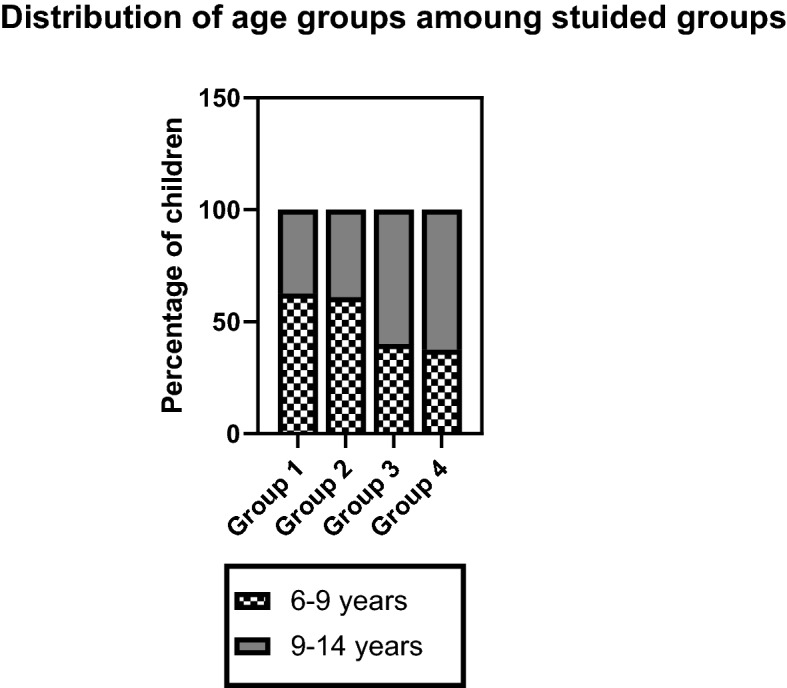
Distribution of age groups amoung studied groups

The sociodemographic characteristics of the study groups are illustrated in Table 1. There was a significant difference between groups regarding age, gender, number and order of siblings in families, motor and speech development delays, socioeconomic status, and the number of devices used to access internet gaming apps. Participants who used internet gaming applications for 1–2 h and those who used internet gaming applications for 3–4 h were the youngest (8.43 ± 2.47, 8.7 ± 2.8, respectively) while participants who used internet gaming applications for 5–6 h and those who used internet gaming applications for more than 6 h were the oldest (9.6 ± 2.51, 9 ± 3.09, respectively)( see Figure 1). There was a higher proportion of males (58.9%) than females (41.1%). The majority of the participants were first-born and had only one sibling. The percentage delay in speech and motor development, as well as the number of devices used to access internet gaming apps, increased with increased hours of use until it reached 6 h, after which the percentage decreased. In terms of socioeconomic level, the group that used internet gaming applications for more than 6 h had the lowest mean socioeconomic score. In all groups, the middle socioeconomic level had the highest proportion, followed by the lower socioeconomic level.

**Table 1:** The sociodemographic data among the studied groups

Variables	Group 1 (N = 320)	Group 2 (N = 120)	Group 3 (N = 92)	Group 4 (N = 32)	Total participants (N = 564)	X^2^ or F Value	P value
Age (mean ± SD)	8.43 ± 2.47	8.7 ± 2.28	9.6 ± 2.51	9 ± 3.09	8.77 ± 2.53	7.34	0.001*
Gender							
Males	176 (55%)	84 (70%)	56 (60.9%)	16 (50%)	332 (58.9%)	9.31	0.02*
Females	144 (45%)	36 (30%)	36 (39.1%)	16 (50%)	232 (41.1%)		
Order of birth							
First	268 (83.8%)	76 (63.3%)	60 (65.2%)	20 (62.5%)	424 (75.2%)	53.77	0.001*
Second	44 (13.8%)	28 (23.3%)	20 (21.7%)	4 (12.5%)	96 (17%)		
Third	4 (1.3%)	4 (3.3%)	8 (8.7%)	4 (12.5%)	20 (3.5%)		
Fourth or more	4 (1.3%)	12 (10%)	4 (4.3%)	4 (12.5%)	24 (4.3%)		
Number of siblings in family							
Only child	52 (16.3%)	4 (3.3%)	26 (17.4%)	12 (37.5%)	84 (14.9%)	42.94	0.0001*
Two children	164 (51.2%)	68 (56.7%)	44 (47.8%)	8 (25%)	284 (50.4%)		
Third	88 (27.5%)	28 (23.3%)	24 (26.1%)	8 (25%)	148 (26.2%)		
Four children or more	16 (5%)	20 (16.7%)	8 (8.7%)	4 (12.5%)	48 (8.5%)		
Delay of speech development	32 (10%)	28 (23.3%)	24 (26.1%)	4 (12.5%)	88 (15.6%)	20.98	0.001*
Delay of motor development	8 (2.5%)	8 (6.7%)	8 (8.7%)	0(0%)	24 (4.3%)	10.007	0.019*
Family history of psychiatric disorders	49 (15.3%)	27 (22.5%)	20 (21.7%)	5 (15.6%)	101 (17.9%)	7.47	0.27
Socioeconomic level (mean ± SD)	235.47 ± 32.05	230.5 ± 31.88	239.5 ± 31.7	217.8 ± 38.6	234.08 ± 32.64	4.2	0.006*
High level	16 (5%)	0 (0%)	8 (3.3%)	0 (0%)	24 (4.3%)	15.1	0.019*
Middle level	248 (77.5%)	92 (76.7%)	72 (78.3%)	24 (75%)	436 (77.3%)		
Low level	56 (17.5%)	28 (23.3%)	12 (13%)	8 (25%)	104 (18.4%)		
Number of devices using Internet gaming apps (mean ± SD)	1.24 ± 0.48	1.53 ± 0.7	1.9 ± 0.7	1.7 ± 0.6	1.44 ± 0.64	35.8	0.001*

Group 1 = participants use internet gaming apps for 1–2 h, Group 2 = participants use internet gaming apps for 3–4-h, Group 3 = participants use internet gaming apps for 5–6-h, Group 4 = participants use internet gaming apps for more than 6 h

### The Strengths and Difficulties Questionnaire (SDQ) scale

There was a significant statistical difference between the groups studied on all SDQ subscales. In comparison to other groups, children who used internet gaming applications for more than 6 h had a higher proportion of abnormal responses on the emotional symptoms and hyperactivity scales (see Table 2).

**Table 2:** The Strengths and Difficulties Questionnaire (SDQ) scale scores among the studied groups

Variables	Group 1 (N = 320)	Group 2 (N = 120)	Group 3 (N = 92)	Group 4 (N = 32)	Total participants (N = 564)	X^2^ value	P value
Emotional symptoms scale							
Normal	104(32.5%)	28 (23.3%)	20 (21.7%)	4 (12.5%)	156 (27.7%)	18.26	0.006*
Borderline	32 (10%)	4 (3.3%)	12 (13%)	4 (12.5%)	52 (9.2%)		
Abnormal	184(57.5%)	88 (73.3%)	60 (65.2%)	24 (75%)	356 (63.1%)		
Conduct symptoms scale							
Normal	228 (71.3%)	52 (43.3%)	48 (52.2%)	20 (62.5%)	348 (61.7%)	41.3	0.001*
Borderline	40 (12.5%)	20 (16.7%)	16 (17.4%)	8 (25%)	84 (14.9%)		
Abnormal	52 (16.3%)	48 (40%)	28 (30.4%)	4(12.5%)	132 (23.4%)		
Hyperactivity scale							
Normal	200 (62.5%)	60 (50%)	52 (56.5%)	16(50%)	328 (58.2%)	20.4	0.002*
Borderline	12 (3.8%)	16 (13.3%)	8 (8.7%)	0(0%)	36 (6.4%)		
Abnormal	108 (33.8%)	44 (36.8%)	32 (34.8%)	16(50%)	200 (35.5%)		
Peer problems scale							
Normal	144 (45%)	52 (43.3%)	28 (30.4%)	4 (12.5%)	228 (40.4%)	60.6	0.001*
Borderline	64 (20%)	20 (16.7%)	20 (21.7%)	24 (75%)	128 (22.7%)		
Abnormal	112 (35%)	48 (40%)	44 (47.8%)	4 (12.5%)	208 (36.9%)		
Prosocial scale							
Normal	248 (77.5%)	68 (56.7%)	68 (73.9%)	28 (87.5%)	412 (73%)	25.3	0.001*
Borderline	32 (10%)	24 (20%)	12 (13%)	4 (12.5%)	72 (12.8%)		
Abnormal	40 (12.4%)	28 (23.3%)	12 (13%)	0 (0%)	80 (14.2%)		
The total difficulties scale							
Normal	32 (10%)	12 (10%)	4 (4.3%)	0(0%)	48 (8.5%)	19.18	0.004*
Borderline	44 (13.8%)	16 (13.3%)	3 (4.3%)	0(0%)	64 (11.3%)		
Abnormal	244 (76.3%)	92 (76.7%)	84 (91.3%)	32 (100%)	452 (80.1%)		

Group 1 = participants use internet gaming apps for 1–2 h, Group 2 = participants use internet gaming apps for 3–4-h, Group 3 = participants use internet gaming apps for 5–6-h, Group 4 = participants use internet gaming apps for more than 6 h

In comparison to the other groups, children who used internet gaming applications for 1–2 h had the lowest percentage prosocial scale.

Participants who used internet gaming apps for more than 6 h all scored abnormally on the total difficulties scale, but none scored abnormally on the prosocial scale. This group also had the lowest percentage of abnormal responses in the conduct subscale and peer problems.

### The Children's Sleep Habits Questionnaire

Table 3 displays the results of the Children's Sleep Habits Questionnaire. There were significant differences in the total bedtime subscale and total morning wake-up scores. Children who used internet gaming applications for more than 6 h had the highest scores in all subscales as well as the total score in the Children's Sleep Habits Questionnaire, while children who used internet gaming applications for 1–2 h had the lowest scores. There was the highest percentage of poor sleep quality (75%) in children who used internet gaming applications for more than 6 h and the lowest percentage in children who used internet gaming applications for 1–2 h (36.7%).

**Table 3:** The Children's Sleep Habits Questionnaire among the studied groups

Variables	Group 1 (N = 320)	Group 2 (N = 120)	Group 3 (N = 92)	Group 4 (N = 32)	Total participants (N = 564)	X^2^ or F value	P value
The Children's Sleep Habits Questionnaire subscales							
Total bedtime subscale	14.9 ± 4.6	15.4 ± 4.1	16.7 ± 4.1	17.3 ± 4.7	16.18 ± 4.49	6.3	0.001*
Total sleep behavior subscales	7.8 ± 7.4	8.6 ± 8.1	9.1 ± 10.6	9.7 ± 8.7	8.89 ± 8.78	0.5	0.66
Total waking during the night subscale	1.5 ± 1.5	1.9 ± 1.7	1.9 ± 1.5	2.3 ± 2.5	1.90 ± 1.73	2.4	0.05
Total morning wakes up subscale	4.3 ± 2.6	5.08 ± 3.1	5.3 ± 2.4	6.7 ± 2.3	5.05 ± 2.93	6.3	0.001*
Total Score of scale	29.9 ± 14.1	32.44 ± 12.2	32.4 ± 12.4	34.3 ± 12.8	32.01 ± 12.79	1.5	0.19
The interpretation of The Children's Sleep Habits Questionnaire							
Goop sleep quality	203 (63.3%)	52 (43.3%)	40 (43.5%)	8 (25%)	264 (46.8%)	20.8	0.001*
Poor sleep quality	117 (36.7%)	68 (56.7%)	52 (56.5%)	24 (75%)	300 (53.2%)		
Sleep time variables							
Night-time sleep duration per hours	8.9 ± 1.4	8.4 ± 1.7	8.3 ± 1.5	9.2 ± 1.6	8.7 ± 1.5	5.6	0.001*
Daytime sleep duration per minute	24.8 ± 40.5	36.5 ± 65.04	14.5 ± 34.7	8.7 ± 13.6	24.7 ± 45.63	5.5	0.001*
Total sleep duration per hours	9.3 ± 1.3	9.1 ± 1.3	8.6 ± 1.2	9.5 ± 1.6	9.2 ± 1.3	8.7	0.001*

Group 1 = participants use internet gaming apps for 1–2 h, Group 2 = participants use internet gaming apps for 3–4-h, Group 3 = participants use internet gaming apps for 5–6-h, Group 4 = participants use internet gaming apps for more than 6 h

There was a significant difference between groups in terms of night-time and daytime sleep duration, as well as total sleep duration per hour. Participants who used internet gaming applications for more than 6 h had a longer night-time sleep duration per hour (9.2 ± 1.6) and a shorter daytime sleep duration per minute (8.7 ± 13.6).

### The Children's Alexithymia Measure (CAM) Scores and the Clinical Evaluation of Emotional Regulation–9

The total score of the Clinical Evaluation of Emotional Regulation–9 and total CAM was lowest in participants who used internet gaming apps for 1–2 h; the highest CAM score occurred in children who used internet gaming apps for more than 6 h (See Table 4).

**Table 4:** The Children’s Alexithymia Measure (CAM) scores and The Clinical Evaluation of Emotional Regulation–9 among the studied groups

Variables	Group 1 (N = 320)	Group 2 (N = 120)	Group 3 (N = 92)	Group 4 (N = 32)	Total participants (N = 564)	F value	P value
The Children’s Alexithymia Measure (CAM) (mean ± SD)	11.43 ± 9.7	12.43 ± 8.8	11.74 ± 9.4	14.13 ± 10.5	11.8 ± 9.5	0.9	0.4
The Clinical Evaluation of Emotional Regulation–9(mean ± SD)	11.5 ± 6.2	14.83 ± 6.4	12.7 ± 4.5	12 ± 5.3	12.5 ± 6.1	9.2	0.001*

Group 1 = participants use internet gaming apps for 1–2 h, Group 2 = participants use internet gaming apps for 3–4-h, Group 3 = participants use internet gaming apps for 5–6-h, Group 4 = participants use internet gaming apps for more than 6 h

### Identification of Possible Risk Factors for the Increased Number of Internet Gaming Apps Used by Children

Table 5 shows the results of univariate linear the regression analysis evaluating the multiple risk factors affecting participants' use of internet gaming apps. Participants with high emotion dysregulation score (p = 0.025), shorter sleep duration (day and night) (p = 0.021), and a delay in morning wake-up (p = 0.005) were more likely to use internet gaming apps. Using internet gaming apps, on the other hand, was associated with a higher number of devices (p = 0.0001), emotional problems (p = 0.002), conduct problems (p = 0.001), hyperactivity difficulties (p = 0.015), peer problems (p = 0.04) and the total difficulties (p = 0.001). Controlling for gender led to the same results (see Additional file 1: table S6 and S7). An increased number of devices and shorter sleep duration (day and night) were associated with more intensive use of internet gaming apps in both genders. (see Additional file 1: table S8 and S9).

**Table 5:** univariate linear Regression model between hours of internet gaming apps and other parameters

Variables	B	Std. Error	Beta	t	P value	95.0% Confidence Interval	
						Lower Bound	Upper Bound
Age	0.053	0.031	0.072	1.717	0.08	− 0.008	0.114
Gender	− 0.102	0.17	− 0.02	− 0.57	0.56	− 0.45	0.24
Number of devices	1.29	0.124	0.402	10.4	0.0001*	1.04	1.53
Total score of socioeconomic scale	− 0.003	0.003	− 0.055	− 1.3	0.19	− 0.009	− 0.002
The Children’s Alexithymia Measure (CAM)	0.009	0.009	0.044	1.033	0.302	− 0.009	0.027
The Clinical Evaluation of Emotional Regulation–9	0.032	0.014	0.094	2.24	0.025*	0.004	0.06
Total nighttime sleep duration	− 0.074	0.057	− 0.055	− 1.309	0.19	− 1.18	0.037
Total daytime sleep duration	− 0.003	0.002	− 0.073	− 1.74	0.082	− 0.007	0.001
Total sleep duration (day and night)	− 0.148	0.064	− 0.097	− 2.31	0.021*	− 0.273	− 0.022
Total bedtime	− 0.018	0.019	− 0.04	− 0.940	0.348	− 0.056	0.020
Total sleep behavior	− 0.003	0.01	− 0.011	− 0.261	0.794	− 0.022	0.017
Total waking during the night	0.028	0.05	0.023	0.557	0.578	− 0.071	0.127
Total morning wakes up	0.084	0.03	0.118	2.82	0.005*	0.026	0.142
Total Score of scale	0.001	0.007	0.009	− 0.210	0.833	− 0.012	0.015
Emotional symptoms scale	0.115	0.036	0.133	3.18	0.002*	0.044	0.186
Conduct symptoms scale	0.172	0.051	0.14	3.34	0.001*	0.071	0.273
Hyperactivity scale	0.074	0.030	0.102	2.44	0.015*	0.014	0.133
Peer problems scale	0.125	0.044	0.120	2.86	0.004*	0.039	0.211
Prosocial scale	− 0.034	0.038	− 0.037	− 0.886	0.37	− 0.11	0.041
The total difficulties scale	0.059	0.015	0.169	4.06	0.001*	0.031	0.088

## Discussion

Among the numerous factors related to problematic online gaming, time spent playing online games has been one of the most controversial [43]. However, most international scientific communities recommend less than 2 h per day as a total screen time for children under 17 years old. Here we investigated the time spent using internet gaming apps in children and its association with behavior problems, sleep problems, alexithymia, and emotional regulation.

In the current study, there was a significant difference between groups in terms of age, gender, order of birth, number of siblings in families, motor and speech development delay, and the number of devices used to access internet gaming apps. Participants in groups 3 and 4, who used internet gaming applications for 5–6 h or more than 6 h, were older than those who used them for 1–2 or 3–4 h. This finding was consistent with a 2-year follow-up study in South Korea, which found that individuals at high risk for IGD were more likely to be older children and spend longer times gaming per day [43]. This could be due to more exposure to the internet in older children who use it for online studying and communication, particularly after the COVID-19 pandemic.

All groups had a higher proportion of boys than girls. A large US study found that problematic internet gaming occurs up to five times more frequently in male children (11.9%) than in females (2.9%)[12], and an Australian study of internet use and electronic gaming by children and adolescents found that only 5.3% of boys did not play electronic games compared to 24.8% of girls [44]. Males have 2.5 times more GD than females, according to a global systematic review [45]. This discrepancy may be caused by the popularity of online games for social networking and other associated activities among females [46], whereas fighting and action games are more popular among boys and may be more engaging when played in a group of peers.

Most participants were first-born and had one sibling with a middle socioeconomic status. Our finding is consistent with previous research conducted among Egyptian university students [47], however it contradicts a study conducted in China, where family structure was unimportant [48]. These disparities could be attributed to factors such as participant age, cultural variations, and sociodemographic factors. Our results in Egypt could be related to the fact that the children's parents are preoccupied with raising a new child, the children may exhibit more extreme behaviours to get their parents' attention, and the first child's feelings of sibling jealousy contributed to the rise in internet gaming app use.

There was a substantial statistical difference between the groups in all SDQ subscales. In Group 4, who used internet gaming applications for more than 6 h, abnormal responses in the emotional symptom scale and the hyperactivity scale were higher than in the other groups. In addition, they had abnormal responses in the total difficulties scale, while none of them had an abnormal response in the prosocial scale. This group also had the highest percentage of abnormal responses in the conduct subscale and peer problems.

This finding was consistent with a survey on the mental health and wellbeing of children and adolescents aged 4–17 years in Australia, where Rikkers and his colleagues investigated the association of internet use and electronic gaming with emotional and behavioral problems. They used the Kessler 10 Psychological Distress Scale (K10) for younger children and the Strengths and Difficulties Questionnaire (SDQ) for older children and adolescents. They found that children who spent more daily time at the weekend playing online games scored high on both K10 and SDQ [44].

Additionally, Italian research on school-aged children found that ADHD patients with IGD presented with more severe symptoms. A binary logistic regression showed that IGD was correlated with the degree of inattention. This may be because the main characteristics of IGD are similar to those of ADHD, including the impulsive urge for quick gratification and the tendency for sensation-seeking activities. This link may be useful in the treatment and management of IGD via ADHD treatment and management techniques [49].

Another review investigating IGD in children found that predisposing comorbidities and health-related consequences, in addition to poor relationships with parents and peers, were commonly observed in children with IGD [1]. Additionally, adolescents in Singapore [50] and Germany [51] were found to have longitudinal correlations between emotional problems and the prevalence of IGD. Korean schoolchildren also showed a stronger correlation between IGD and emotional disorders with follow-up for one year, which was found to be 2.8 times higher than in participants without emotional problems [52]. IGD may result from attempts to utilize online games to self-regulate, escape, or ease unpleasant emotions [53].

The present study also found that participants who used internet gaming apps for 1–2 h had significantly lower mean total scores on the Clinical Evaluation of Emotional Regulation–9 and total CAM. The group of children who used internet gaming apps for more than 6 h had the highest mean of CAM. Furthermore, participants with high score of emotion dysregulation were more likely to spend increased time on online gaming apps. A study that was conducted on middle and high school-aged French adolescents revealed that regular online gamers, who played significantly more hours than irregular gamers, regulated their emotions more than irregular gamers did. They also felt more intensely. But regular gamers displayed emotions less than irregular players. Also, frequent gamers had higher alexithymia levels than irregular gamers [54].

Online addictive behaviours may have an impact on how emotions are regulated by strengthening control of emotions, obtaining online social validation, and compensating for disadvantages in the real life [55]. It has been hypothesized that excessive online behaviours are indicators of a range of diseases, including depression [56]. Playing an excessive number of online hours may thus be a technique to treat pre-existing depressed psychopathology, which itself might subsequently provoke additional symptomatology. Therefore, integrating therapy to handle children's emotional problems may have additional advantages for preventing IGD in children and adolescents [57].

The total bedtime subscale and total morning wake-up scores were significantly different between groups, with children who used internet gaming applications for more than 6 h scoring highest in all subscales as well as the total score in the Children's Sleep Habits Questionnaire. For example, children who used internet gaming applications for more than 6 h had the worst sleep quality (75%). Furthermore, those who used internet gaming applications for more than 6 h had longer night-time sleep duration per hour and a shorter daytime sleep duration per minute. Also, participants with shorter sleep duration (day and night) and delay in morning wake up more likely to use internet gaming apps.

Our findings matched those of a systematic review of seven studies on the relationship between internet gaming and sleep problems which found that problematic or addictive gaming, particularly massively multiplayer online role-playing games, is associated with sleep problems, including poorer sleep quality and shorter sleep duration [58]. Another systematic review discussed the association between screen time and sleep patterns among school-aged children and adolescents. It found associations between screen time and reduced sleep quality, longer sleep onset latency, delayed bedtime, shorter total sleep time and increased daytime tiredness. In addition, 86% of studies found an association between video game use and abnormal sleep patterns [59]. The proposed mechanisms for sleep disturbance included displacement of sleep time, psychological stimulation, light exposure, and increased physiological alertness [17].

Several limitations must be considered when interpreting our findings. First, this study did not assess scholastic achievement in children who use internet gaming applications excessively. Second, the cross-sectional research design of the current study may limit the ability to draw causal conclusions between video gaming addiction and related characteristics. Thirdly, more information about the nature and content of games, as well as player types was needed to conduct a more thorough investigation of IGA's impact on psychiatric issues. Finally, possible negative feelings of parents about their children’s internet gaming app usage may bias answers to the questionnaires.

## Conclusions

An increased number of hours pent using internet gaming applications was associated with more psychiatric problems, sleep disturbance, alexithymia, and emotion dysregulation. Excessive use of internet gaming apps made children more susceptible to hyperactivity and peer problems. Participants with excessive use of internet gaming apps were more likely to have a high socioeconomic status, high alexithymia issues, shorter daytime sleep duration, and a morning wake-up delay.

## Supplementary Information

**Additional file 1: Table S6.** univariate linear Regression model between hours of internet gaming apps and other parameters in male. **Table S7.** Univariate linear Regression model between hours of internet gaming apps and other parameters in female. **Table S8.** Multi linear Regression model between hours of internet gaming apps and other parameters in male. **Table S9.** Multi linear Regression model between hours of internet gaming apps and other parameters in female

## Data Availability

All data generated or analyzed during this study are available from corresponded on request.

## Abbreviations

IGD: Internet gaming disorder
ICD-11: The International Classification of Diseases-11th revision
CSHQ-A: The Children's Sleep Habits Questionnaire Abbreviated
SDQ: The strengths and difficulties questionnaire
CAM: The Children’s Alexithymia Measure
SNAP: Swanson Nolan and Pelham
