# Supplementary material for: Relationship between time spent playing internet gaming apps and behavioral problems, sleep problems, alexithymia, and emotion dysregulations in children: a multicentre study
Source: Child Adolesc Psychiatry Ment Health. 2022 Aug 16;16:67. doi: 10.1186/s13034-022-00502-w (PMC9380675; doi:10.1186/s13034-022-00502-w)
Supplement: Supplementary file 1 — Additional file 1: Table S6. univariate linear Regression model between hours of internet gaming apps and other parameters in male. Table S7. Univariate linear Regression model between hours of internet gaming apps and other parameters in female. Table S8. Multi linear Regression model between hours of internet gaming apps and other parameters in male. Table S9. Multi linear Regression model between hours of internet gaming apps and other parameters in female [file 13034_2022_502_MOESM1_ESM.docx]

**Supplement material**

**Table 6: univariate linear Regression model between hours of internet gaming apps and other parameters in male**


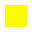


| **Variables** | **B** | **Std. Error** | **Beta** | **t** | **P value** | **95.0% Confidence Interval** | |
| --- | --- | --- | --- | --- | --- | --- | --- |
|  |  |  |  |  |  | **Lower Bound** | **Upper Bound** |
| **Age** | 0.053 | 0.031 | 0.072 | 1.717 | 0.086 | -0.008 | 0.114 |
| **Number of devices** | 1.291 | 0.124 | 0.402 | 10.405 | 0.0001* | 1.047 | 1.535 |
| **Total score of socioeconomic scale** | -0.003 | 0.003 | -0.055 | -1.305 | 0.192 | -.009 | 0.002 |
| **The Children’s Alexithymia Measure (CAM)** | 0.009 | 0.009 | 0.044 | 1.033 | 0.302 | -0.009 | 0.027 |
| **The Clinical Evaluation of Emotional Regulation–9** | 0.032 | 0.014 | 0.094 | 2.243 | 0.025* | 0.004 | 0.060 |
| **Total nighttime sleep duration** | -0.074 | 0.057 | -0.055 | -1.309 | 0.191 | -0.186 | 0.037 |
| **Total daytime sleep duration** | -0.003 | 0.002 | -0.073 | -1.740 | 0.082 | -0.007 | 0.0001 |
| **Total sleep duration (day and night)** | -0.148 | 0.064 | -0.097 | -2.312 | 0.021* | -0.273 | -0.022 |
| Total bedtime | -0.018 | 0.019 | -0.040 | -0.940 | 0.348 | -0.056 | 0.020 |
| Total sleep behavior | -0.003 | 0.010 | -0.011 | -0.261 | 0.794 | -0.022 | 0.017 |
| Total waking during the night | 0.028 | 0.050 | 0.023 | 0.557 | 0.578 | -0.071 | 0.127 |
| Total morning wakes up | 0.084 | 0.030 | 0.118 | 2.828 | 0.005* | 0.026 | 0.142 |
| Total Score of scale | 0.001 | 0.007 | 0.009 | 0.210 | 0.833 | -0.012 | 0.015 |
| **Emotional symptoms scale** | 0.115 | 0.036 | 0.133 | 3.182 | 0.002* | 0.044 | 0.186 |
| **Conduct symptoms scale** | 0.172 | 0.051 | 0.140 | 3.349 | 0.001* | 0.071 | 0.273 |
| **Hyperactivity scale** | 0.074 | 0.030 | 0.102 | 2.442 | 0.015* | 0.014 | 0.133 |
| **Peer problems scale** | 0.125 | 0.044 | 0.120 | 2.867 | 0.004* | 0.039 | 0.211 |
| **Prosocial scale** | -0.034 | 0.038 | -0.037 | -0.886 | 0.376 | -0.110 | 0.041 |
| **The total difficulties scale** | 0.059 | 0.015 | 0.169 | 4.069 | 0.0001* | 0.031 | 0.088 |

**Table 7: univariate linear Regression model between hours of internet gaming apps and other parameters in female**


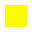


| **Variables** | **B** | **Std. Error** | **Beta** | **t** | **P value** | **95.0% Confidence Interval** | |
| --- | --- | --- | --- | --- | --- | --- | --- |
|  |  |  |  |  |  | **Lower Bound** | **Upper Bound** |
| **Age** | 0.054 | 0.031 | 0.074 | 1.747 | 0.081 | -0.007 | 0.115 |
| **Number of devices** | 1.290 | 0.124 | 0.402 | 10.382 | 0.0001* | 1.046 | 1.534 |
| **Total score of socioeconomic scale** | -0.003 | 0.003 | -0.053 | -1.259 | 0.208 | -0.009 | 0.002 |
| **The Children’s Alexithymia Measure (CAM)** | 0.009 | 0.009 | 0.042 | 0.983 | 0.326 | -0.009 | 0.027 |
| **The Clinical Evaluation of Emotional Regulation–9** | 0.032 | 0.014 | 0.093 | 2.180 | 0.030* | 0.003 | 0.060 |
| **Total nighttime sleep duration** | -0.074 | 0.057 | -0.055 | -1.304 | 0.193 | -0.186 | 0.038 |
| **Total daytime sleep duration** | -0.003 | 0.002 | -0.073 | -1.726 | 0.085 | -0.007 | 0.0001 |
| **Total sleep duration (day and night)** | -0.147 | 0.064 | -0.097 | -2.301 | 0.022* | -0.273 | -0.022 |
| Total bedtime | -0.018 | 0.019 | -0.039 | -0.915 | 0.361 | -0.056 | 0.020 |
| Total sleep behavior | -0.003 | 0.010 | -0.011 | -0.252 | 0.801 | -0.022 | 0.017 |
| Total waking during the night | 0.032 | 0.051 | 0.027 | 0.631 | 0.528 | -0.068 | 0.132 |
| Total morning wakes up | 0.085 | 0.030 | 0.120 | 2.862 | 0.004* | 0.027 | 0.143 |
| Total Score of scale | 0.002 | 0.007 | 0.010 | 0.242 | 0.809 | -0.012 | 0.015 |
| **Emotional symptoms scale** | 0.114 | 0.036 | 0.132 | 3.136 | 0.002* | 0.043 | 0.186 |
| **Conduct symptoms scale** | 0.173 | 0.051 | 0.140 | 3.360 | 0.001* | 0.072 | 0.274 |
| **Hyperactivity scale** | 0.074 | 0.031 | 0.102 | 2.371 | 0.018* | 0.013 | 0.134 |
| **Peer problems scale** | 0.124 | 0.044 | 0.119 | 2.816 | 0.005* | 0.038 | 0.211 |
| **Prosocial scale** | -.032 | 0.039 | -0.035 | -0.836 | 0.403 | -0.108 | 0.044 |
| **The total difficulties scale** | 0.059 | 0.015 | 0.169 | 4.024 | 0.0001* | 0.030 | 0.088 |

**Table 8: Multi linear Regression model between hours of internet gaming apps and other parameters in male**


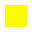


| **Variables** | **B** | **Std. Error** | **Beta** | **t** | **P value** | **95.0% Confidence Interval** | |
| --- | --- | --- | --- | --- | --- | --- | --- |
|  |  |  |  |  |  | **Lower Bound** | **Upper Bound** |
| **Number of devices** | 1.206 | 0.127 | 0.375 | 9.497 | 0.0001* | 0.956 | 1.455 |
| **The Clinical Evaluation of Emotional Regulation–9** | -0.017 | 0.020 | -0.051 | -0.850 | 0.396 | -0.057 | 0.023 |
| **Total sleep duration (day and night)** | -0.161 | 0.060 | -0.106 | -2.706 | 0.007* | -0.279 | -0.044 |
| **Total morning wakes up** | 0.047 | 0.029 | 0.066 | 1.643 | 0.101 | -0.009 | 0.103 |
| **Emotional symptoms scale** | -0.011 | 0.064 | -0.013 | -0.174 | 0.862 | -0.138 | 0.115 |
| **Conduct symptoms scale** | 0.078 | 0.073 | 0.063 | 1.057 | 0.291 | -0.067 | 0.222 |
| **Hyperactivity scale** | -0.048 | 0.061 | -0.067 | -0.785 | 0.433 | -0.169 | 0.072 |
| **Peer problems scale** | 0.053 | 0.053 | 0.051 | 1.014 | 0.311 | -0.050 | 0.156 |
| **The total difficulties scale** | 0.043 | 0.045 | 0.123 | 0.955 | 0.340 | -0.045 | 0.131 |

**Table 9: Multi linear Regression model between hours of internet gaming apps and other parameters in female**

| **Variables** | **B** | **Std. Error** | **Beta** | **t** | **P value** | **95.0% Confidence Interval** | |
| --- | --- | --- | --- | --- | --- | --- | --- |
|  |  |  |  |  |  | **Lower Bound** | **Upper Bound** |
| **Number of devices** | 1.206 | 0.127 | 0.375 | 9.487 | 0.0001* | 0.956 | 1.455 |
| **The Clinical Evaluation of Emotional Regulation–9** | -0.018 | 0.021 | -0.054 | -0.893 | 0.372 | -0.059 | 0.022 |
| **Total sleep duration (day and night)** | -0.161 | 0.060 | -0.106 | -2.692 | 0.007* | -0.278 | -0.043 |
| **Total morning wakes up** | 0.048 | 0.029 | 0.068 | 1.667 | 0.096 | -0.009 | 0.104 |
| **Emotional symptoms scale** | -0.012 | 0.065 | -0.014 | -0.184 | 0.854 | -0.139 | 0.115 |
| **Conduct symptoms scale** | 0.082 | 0.075 | 0.067 | 1.107 | 0.269 | -0.064 | 0.229 |
| **Hyperactivity scale** | -0.052 | 0.062 | -0.072 | -0.837 | 0.403 | -0.175 | 0.070 |
| **Peer problems scale** | 0.051 | 0.053 | 0.049 | 0.965 | 0.335 | -0.053 | 0.155 |
| **The total difficulties scale** | 0.044 | 0.045 | 0.125 | 0.972 | 0.332 | -0.045 | 0.132 |
